# Automatic detection of hippocampal sclerosis in patients with epilepsy

**DOI:** 10.1111/epi.18514

**Published:** 2025-06-21

**Authors:** Marcus Belke, Felix Zahnert, Mirja Steinbrenner, Mustafa Halimeh, Gadi Miron, Panagiota‐Eleni Tsalouchidou, Louise Linka, Boris Keil, Andreas Jansen, Vincent Möschl, André Kemmling, Christopher Nimsky, Felix Rosenow, Katja Menzler, Susanne Knake

**Affiliations:** ^1^ Department of Neurology, Epilepsy Center Hessen Philipps Universität Marburg Marburg Germany; ^2^ Landes‐Offensive zur Entwicklung Wissenschaftlich‐ökonomischer Exzellenz (LOEWE) Research Cluster for Advanced Medical Physics in Imaging and Therapy Technische Hochschule (TH)‐Mittelhessen University of Applied Sciences Giessen Germany; ^3^ Center for Personalized Translational Epilepsy Research, Goethe Universität Frankfurt Frankfurt am Main Germany; ^4^ Department of Neurology with Experimental Neurology Computational Neurology, Charité–Universitätsmedizin Berlin, corporate member of Freie Universität Berlin and Humboldt‐Universität zu Berlin Berlin Germany; ^5^ Berlin Institute of Health at Charité–Universitätsmedizin Berlin Berlin Germany; ^6^ Department of Life Science Engineering, Institute of Medical Physics and Radiation Protection TH‐Mittelhessen University of Applied Sciences Giessen Germany; ^7^ Department of Diagnostic and Interventional Radiology Philipps Universität Marburg Marburg Germany; ^8^ Center for Brain, Mind, and Behavior, Philipps Universität Marburg Marburg Germany; ^9^ Department of Psychiatry Philipps Universität Marburg Marburg Germany; ^10^ Department of Neuropathology Philipps Universität Marburg Marburg Germany; ^11^ Department of Neuroradiology Philipps Universität Marburg Marburg Germany; ^12^ Department of Neurosurgery Philipps Universität Marburg Marburg Germany; ^13^ Department of Neurology, Center of Neurology and Neurosurgery Goethe Universität Frankfurt, Epilepsy Center Frankfurt Rhine‐Main Frankfurt Germany

**Keywords:** FreeSurfer, hippocampal sclerosis, hippocampal subfield segmentation, machine learning, MRI

## Abstract

**Objective:**

This study was undertaken to develop and validate an automatic, artificial intelligence‐enhanced software tool for hippocampal sclerosis (HS) detection, using a variety of standard magnetic resonance imaging (MRI) protocols from different MRI scanners for routine clinical practice.

**Methods:**

First, MRI scans of 36 epilepsy patients with unilateral HS and 36 control patients with epilepsy of other etiologies were analyzed. MRI features, including hippocampal subfield volumes from three‐dimensional (3D) magnetization‐prepared rapid acquisition gradient echo (MPRAGE) scans and fluid‐attenuated inversion recovery (FLAIR) intensities, were calculated. Hippocampal subfield volumes were corrected for total brain volume and *z*‐scored using a dataset of 256 healthy controls. Hippocampal subfield FLAIR intensities were *z*‐scored in relation to each subject's mean cortical FLAIR signal. Additionally, left–right ratios of FLAIR intensities and volume features were obtained. Support vector classifiers were trained on the above features to predict HS presence and laterality. In a second step, the algorithm was validated using two independent, external cohorts, including 118 patients and 116 controls in sum, scanned with different MRI scanners and acquisition protocols.

**Results:**

Classifiers demonstrated high accuracy in HS detection and lateralization, with slight variations depending on the input image availability. The best cross‐validation accuracy was achieved using both 3D MPRAGE and 3D FLAIR scans (mean accuracy = 1.0, confidence interval [CI] = .939–1.0). External validation of trained classifiers in two independent cohorts yielded accuracies of .951 (CI = .902–.980) and .889 (CI = .805–.945), respectively. In both validation cohorts, the additional use of FLAIR scans led to significantly better classification performance than the use of MPRAGE data alone (*p* = .016 and *p* = .031, respectively). A further model was trained on both validation cohorts and tested on the former training cohort, providing additional evidence for good validation performance. Comparison to a previously published algorithm showed no significant difference in performance (*p* = 1).

**Significance:**

The method presented achieves accurate automated HS detection using standard clinical MRI protocols. It is robust and flexible and requires no image processing expertise.

**Key points**

- Automated and robust detection of hippocampal sclerosis.
- Compatible with clinical standard MRI scans from different scanners.
- Easy‐to‐use method that might facilitate the early detection of hippocampal sclerosis, especially in settings with limited neuroradiological expertise.

## 1 INTRODUCTION

Hippocampal sclerosis (HS) is the most common pathological finding in patients with focal epilepsy who undergo epilepsy surgery (36.4%).^1^, ^2^ In postmortem series, HS could be identified in 30.5%–45% of all patients with epilepsy syndromes.^3^ Although HS indicates poor response to antiseizure medication, these patients often achieve favorable postoperative outcome following epilepsy surgery.^4^, ^5^, ^6^

Despite its clinical significance, accurately detecting HS using conventional MRI remains challenging due to protocol variations, diverse acquisition parameters, and varying image quality.^7^ The diagnosis currently depends on radiological expertise,^8^ yet even experienced neuroradiologists and epileptologists misidentify HS in 10%–36% of cases.^9^ To address this clinical need, an automated detection tool for HS would be invaluable. Several studies have proposed various methods, including fluid‐attenuated inversion recovery (FLAIR) signal normalization and quantitative T2 mapping.^10^, ^11^ However, existing methods often rely on commercial software^12^, ^13^ or may be difficult to apply in a clinical setting.^14^

In this study, we addressed prior limitations by presenting a novel, flexible, and user‐friendly software tool for automated detection of HS, based on commonly used magnetic resonance imaging (MRI) acquisitions. Our solution accommodates various MRI scans with various acquisition protocols at different field strengths and can be implemented easily without further clinical information and without reliance on commercial software. It offers an efficient solution for detecting HS, particularly in clinical settings where neuroradiological expertise is limited.

## 2 MATERIALS AND METHODS

### 2.1 MRI acquisition

The training (Marburg) cohort underwent MRI scanning using several Siemens MRI scanners systems (3‐T Trio, 1.5‐T Avanto, and 1.5‐T Espree) at the Epilepsy Center Hessen (Marburg, Germany). All subjects completed a three‐dimensional (3D) magnetization‐prepared rapid acquisition gradient echo (MPRAGE) scan (voxel dimension = 1 × 1 × 1 mm, repetition time [TR] = 1.9–2.17 ms, echo time [TE] = 2.26–3.14 ms, inversion time [TI] = 900–1100 ms, flip angle [FA] = 9°–15°). Furthermore, 59 subjects obtained 3D FLAIR scans (voxel dimension = 1 × 1 × 1 mm, TR = 5–6 ms, TE = 358–394 ms, TI = 1800–2200 ms, FA = 120°), and 21 subjects were scanned using a coronal 2D FLAIR scan with ≤4‐mm slice thickness (TR = 9–15 ms, TE = 87–93 ms, TI = 2500–2897 ms, FA = 130–150°).

MRI scans from the first validation cohort were acquired at the Charité Epilepsy Center (Berlin, Germany) using different 3‐T MRI scanners and protocols. The evaluation of the developed software tool utilized 3D MPRAGE and coronal 2D FLAIR scans with ≤4‐mm slice thickness, as well as 3D FLAIR scans, which were only available in a limited number of patients (3D scans in 17 patients and 2D scans in 73 patients).

Detailed acquisition parameters are provided in Tables S1‐S3.

The second validation cohort was generated by including patients with HS and healthy controls from the recently published, open‐source Imaging Database for Epilepsy and Surgery (IDEAS) dataset.^15^ All patients with HS and all healthy controls with a 3D T1 and a FLAIR scan with ≤4‐mm slice thickness were included (3D scans in 114 subjects and 2D scans in 30 subjects).

Details of the scanners used and acquisition parameters can be found in the original publication.^15^

### 2.2 Reference cohort for correcting subfield volumes

To generate *z*‐scores for subfield volumes while correcting for individual brain size, we utilized a reference cohort of 256 healthy subjects. These control subjects underwent 3D MPRAGE scans (voxel dimension = 1 × 1 × 1 mm, TR = 1900 ms, TE = 2.52 ms, TI = 900 ms, FA = 9°) using a 3‐T Trio scanner (Siemens) at the Center for Brain Imaging in Marburg, Germany, as previously described.^16^

### 2.3 MRI preprocessing

All 3D MPRAGE scans were analyzed using the FreeSurfer image analysis suite (v7.4.1, http://surfer.nmr.mgh.harvard.edu) by implementing the recon‐all processing stream,^17^, ^18^, ^19^, ^20^, ^21^, ^22^, ^23^, ^24^, ^25^, ^26^ following our established protocols.^16^ This methodology has previously demonstrated robust test–retest reliability, across different MRI scanner manufacturers and field strengths.^27^, ^28^ In addition, hippocampal subfields were segmented using FreeSurfer, utilizing a probabilistic atlas of hippocampal anatomy, derived from a combination of a T1‐weighted in vivo MRI dataset and 15 ex vivo MRI scans (.13‐mm isotropic resolution), with manually delineated hippocampal subfields (https://surfer.nmr.mgh.harvard.edu/fswiki/SubregionSegmentation).^29^

FLAIR scans were coregistered to the MPRAGE scan using a boundary‐based method,^30^ and bias field correction, using ANTS N4 bias field correction,^31^ was applied.

All scans and results were thoroughly inspected to ensure absence of artifacts and segmentation errors.

### 2.4 Image analysis and feature selection

#### 2.4.1 Volume feature calculation

Hippocampal subfield volumes were corrected for the total brain size using an established covariance approach.^16^, ^32^ This process involved calculating slopes of the linear regression between each subfield volume and the total brain volume in the reference cohort. Subsequently, the volumes of the respective subfields of each subject were corrected, using the following equation:

$${\mathrm{Vol}}_{\text{corr}}={\mathrm{Vol}}_{\text{orig}}-\beta \;\left({\mathrm{TBV}}_{\text{orig}}-{\mathrm{TBV}}_{\text{mean}}\right)$$

where Vol_corr_ represents the corrected volume, Vol_orig_ the original volume of the structure, β the slope of the linear regression, TBV_orig_ the subject's appropriate total brain volume, and TBV_mean_ the mean total brain volume for all control subjects. For the total brain volume, the measure BrainSegVolNotVent in FreeSurfer was used. We calculated *z*‐scores for each corrected subfield volume using the following equation:

$$z=\left({\mathrm{Vol}}_{\text{corr}}-\mu_{\mathrm{NC}}\right)/\sigma_{\mathrm{NC}}$$

where *z* represents the *z*‐score, Vol_corr_ the corrected volume of the structure, *μ*_*NC*_ the mean, and *σ*_NC_ the SD of the structure volume across the reference cohort. For further analysis, only negative *z*‐scores were used and inverted as a marker of atrophy.

Additionally, we computed left–right ratios by dividing each subfield's corrected volume by its contralateral counterpart, to obtain a supplementary feature.

#### 2.4.2 FLAIR feature calculation

The coregistered, bias field‐corrected FLAIR images were resampled to subfield space with a .333‐mm isotropic resolution. Voxelwise FLAIR intensities were *z*‐scored as follows. First, the mean and the SD of FLAIR intensities across the whole cortex were calculated. The voxelwise *z*‐score was then computed using the following equation:

$$z=\left(I_{\text{Voxel}\_\text{Subfield}}-\mu_{\text{Cortex}}\right)/\sigma_{\text{Cortex}}$$

where *z* represents the *z*‐score, *I*_Voxel_Subfield_ the FLAIR intensity of the respective subfield voxel, *μ*_Cortex_ the mean, and σ_Cortex_ the SD of the cortical intensity.

For quantifying hyperintensity, we summed voxelwise *z*‐scores > 1 within each subfield and normalized by the total voxel count to obtain a relative *z*‐score. Although we evaluated multiple *z*‐score thresholds for potential HS detection in the training (Marburg) cohort, models based on FLAIR features with a *z*‐score threshold of 1 demonstrated optimal performance.

Additionally, we derived left–right intensity ratios by dividing each subfield's relative *z*‐score by its contralateral counterpart as a supplementary feature.

#### 2.4.3 Features and feature selection

Six subfields were excluded from the analysis due to low cross‐group variability and no hypothesized involvement in HS pathology (hippocampal (HP) fissure, fimbria, hippocampus–amygdala transition area, presubiculum head/body and parasubiculum). The remaining 16 subfields were used for further computations (CA1 head/body, CA3 head/body, CA4 head/body, subiculum head/body, molecular layer (ML) head/body, granule cell layer (GC) and dentate gyrus (DG) head/body, hippocampal tail, whole hippocampal head/body, whole hippocampus).

For each subfield, four features (volume *z*‐score, volume left–right ratio, FLAIR relative *z*‐core, and FLAIR left–right‐ratio) were computed, resulting in 128 features overall (2 hemispheres × 4 features × 16 subfields).

An additional dataset based only on the MPRAGE scan was generated for training of a separate model. This approach utilized two features per subfield for both hemispheres (volume *z*‐core, volume left–right ratio), resulting in 64 features (2 hemispheres × 2 features × 16 subfields) for subsequent machine learning. This additional dataset served to evaluate the potential added value of an accompanying FLAIR scan and provides an alternative model for cases where only a 3D MPRAGE scan is available.

Labeling of subjects as “HS” or “no HS” was based on histopathology where available. In cases without available histopathology, neuroradiology reports were used.

### 2.5 Machine learning

A support vector machine (SVM)^33^ was trained for each feature dataset using the Python library scikit‐learn version 1.5.1 (https://scikit‐learn.org/stable)^34^ with a linear kernel and standard parameters.

Based on normalized volumes and FLAIR intensities, resulting in 128 features extracted from each individual's bilateral hippocampi (see Section 2.4.3), an SVM with a three‐class classification procedure was generated to classify each patient as having either no HS, left HS, or right HS. Additionally, a three‐class classification SVM was generated using only the normalized volumes obtained from the 3D MPRAGE scans, resulting in 64 features extracted from each individual's bilateral hippocampi (see Section 2.4.3).

For the validation of the SVM on the training (Marburg) set, we performed a k‐fold cross‐validation procedure with 10 splits for the 3D FLAIR data and five splits for the 2D FLAIR data, as 2D FLAIR was only available in 22 subjects. The mean model accuracy along with Clopper–Pearson confidence intervals (CIs) were obtained. To allow for computation of metrics that evaluate analyses of binary targets (area under the curve (AUC) of the receiver operating characteristic (ROC) curve, sensitivity, specificity, and F1 score), this analysis was followed by a binary classification for each hemisphere separately (HS vs. no HS per hemisphere, instead of the above three‐class classification for the entire brain).

### 2.6 External validation of the trained model

The trained SVMs were integrated into an intuitive Python software tool, which is also available as a docker image, accessible via shell command or with a graphical web interface.

To evaluate the generalizability of the proposed algorithm, an external validation cohort of 90 patients from the epilepsy center of the Charité–Universitätsmedizin Berlin and a second validation cohort of 144 subjects from the recently published, open source IDEAS dataset^15^ were analyzed. The software tool, including the 3D FLAIR and the volume‐trained support vector classifiers, processed raw images converted to nifti format, and executed automatic analysis for each subject. The same metrics as mentioned above were computed for each dataset, and McNemar exact test was used to compare the results using volume data alone and using FLAIR data additionally. Patients were labeled as HS based on their postoperative histopathological report in all cases of validation dataset 2 (IDEAS) and in the majority of cases of the Berlin cohort. In the latter, labeling of most control patients was also conducted based on histopathology. If no histopathological report was available, radiological reports were used for labeling cases as “HS” or “no HS.”

### 2.7 Comparison with an established algorithm

We compared the performance of models trained with our presented method with the performance of a recently published and highly promising classifier.^35^

Therefore, support vector classifiers were trained on the pooled data of the Berlin and IDEAS cohorts and were first evaluated via 10‐fold cross‐validation. Here, a mixture of 2D and 3D FLAIR data in addition to MPRAGE scans had been used to include all subjects from the Berlin and IDEAS cohorts for training. This newly trained model was then tested on the Marburg cohort (now as unseen test cohort), as described before.

For comparison, the recently published Automated and Interpretable Detection of Hippocampal Sclerosis (AID‐HS) model^35^ was then tested on the Marburg cohort, and the same performance measures were obtained as described above. Performance of both classifiers on these unseen data was compared using McNemar exact test.

Selection of our original training cohort (Marburg) as the test cohort was necessary for this analysis, as the AID‐HS algorithm required the user to provide patient age as input. This was neither available for Berlin nor for IDEAS cohort data.

### 2.8 Software description

Classifiers that had been trained on the entire training (Marburg) cohort were integrated into a flexible software tool. The model trained on both validation sets from Section 2.7 is optionally also available within the software. This tool runs on multiple operating systems (Linux, Windows, MacOS) and can be executed directly via Python in the Linux shell, in the Windows subsystem for Linux, or in the Darwin shell on MacOS. Alternatively, a docker image featuring an easy‐to‐use graphical web interface is available, enabling deployment on any docker‐supported platform. This ensures broad accessibility and dissemination without commercial software dependencies.

The developed software tool automates all image processing steps from the raw level onward, eliminating the need for image processing expertise, and features a graphical user interface. All necessary software, such as FreeSurfer, is included in the docker container. The processing time without FreeSurfer's recon‐all pipeline is approximately 1.5 min. Including FreeSurfer's image processing extends the runtime to approximately 135 min (depending on the hardware, here Intel Core i7‐8700K CPU with Linux operating system).

Beyond classification results, the software tool generates a list of the computed raw *z*‐scores, facilitating individual visual review of the classification features.

## 3 RESULTS

### 3.1 Patient characteristics

Clinical characteristics of training and validation cohorts are summarized in Table 1. Among the 14 patients in the training (Marburg) cohort with radiologically diagnosed HS who underwent epilepsy surgery, histopathology was available in 13 cases, all with fragmented and sometimes incomplete tissue; therefore, the diagnosis of HS according to International League Against Epilepsy criteria^7^ was often difficult. Neuronal loss within the hippocampus indicating HS was identified in seven patients. In two patients, the diagnosis of probable HS was made. In four patients with radiologically diagnosed HS, tissue quality precluded definitive histological evaluation of HS. The same limitations were present in the control group.

**TABLE 1:** Clinical characteristics of the training and validation cohorts.

Training cohort (Marburg) characteristics	Overall, *n* = 72	Imaging diagnosis of HS, *n* = 36	Controls, *n* = 36
Sex, *n* (%)			
Male	26 (36)	13 (36)	13 (36)
Female	46 (64)	23 (64)	23 (64)
Age, years, mean (SD)	37.8 (14.5)	38.7 (13.6)	36.9 (15.5)
Presence of HS, *n* (%)	36 (50)	36 (100)	–
Left HS	18 (25)	18 (50)	–
Right HS	18 (25)	18 (50)	–
Epilepsy classification, *n* (%)			
Left TLE	23 (32)	18 (50)	5 (14)
Right TLE	20 (28)	18 (50)	2 (6)
Left FLE	3 (4)	–	3 (8)
Right FLE	2 (3)	–	2 (6)
FLE, laterality unclear	1 (1)	–	1 (3)
Hypothalamic hamartoma	2 (3)	–	2 (6)
GGE	17 (24)	–	17 (47)
Unclear	4 (6)	–	4 (11)
Epilepsy surgery, *n* (%)	22 (31)	14 (39)	8 (22)
Histopathology available	21 (29)	13 (36)	7 (19)
Neuronal loss suggesting HS	7 (10)	7 (19)	–
“Probable HS” according to ILAE^7^	2 (3)	2 (6)	–
Histopathological diagnosis of HS not possible based on the available material	4 (6)	4 (11)	–
Extratemporal surgery	2 (3)	–	2 (6)
Temporal resection without evidence of HS	6 (8)	–	6 (17)

First validation cohort (Berlin) characteristics	Overall, *n* = 90	Imaging diagnosis of HS, *n* = 42	Controls, *n* = 48
Sex, *n* (%)			
Male	47 (52)	18 (43)	29 (60)
Female	43 (48)	24 (57)	19 (40)
Presence of HS, *n* (%)	42 (47)	42 (100)	–
Left HS	27 (30)	27 (64)	–
Right HS	15 (17)	15 (36)	–
Temporal epilepsy without HS, *n* (%)	48 (53)	–	48 (100)
Left	25 (28)	–	25 (52)
Right	21 (23)	–	21 (44)
Bilateral	1 (1)	–	1 (2)
Unclear	1 (1)	–	1 (2)
Epilepsy surgery, *n* (%)	85 (94)	37 (88)	48 (100)
Histopathology available, *n* (%)	77 (86)	35 (83)	42 (88)
Histopathological diagnosis of HS, *n* (%)	35 (39)	35 (83)	–
No HS, *n* (%)	42 (47)	–	42 (88)

Second validation cohort (IDEAS) characteristics	Overall, *n* = 144	Diagnosis of HS, *n* = 76	Controls, *n* = 68
Sex, *n* (%)			
Male	57 (40)	32 (42)	25 (37)
Female	87 (60)	44 (58)	43 (63)
Presence of HS, *n* (%)	76 (53)	76 (100)	–
Left HS	41 (29)	41 (54)	–
Right HS	35 (24)	35 (46)	–
Healthy controls, *n* (%)	68 (47)	–	68 (100)
Epilepsy surgery, *n* (%)	76 (53)	76 (100)	–
Histopathology available, *n* (%)	76 (53)	76 (100)	–
Histopathological diagnosis of HS, *n* (%)	76 (53)	76 (100)	–

*Note*: Controls within the training cohort had epilepsies of various etiologies (FLE, GGE, TLE). Controls in the first validation cohort all had TLE; controls in the second validation cohort were healthy participants.

Abbreviations: FLE, frontal lobe epilepsy; GGE, genetic generalized epilepsy; HS, hippocampal sclerosis; ILAE, International League Against Epilepsy; TLE, temporal lobe epilepsy.

In the first validation cohort (Berlin), among the patients with HS, 35 of 37 surgical patients had available histopathology, and HS was identified in all patients. One patient had received a histopathological diagnosis of HS that had been missed on clinical MRI. The control group of the first validation cohort consisted of patients with temporal lobe epilepsy; histopathology was available in 42 of 48 surgical patients, and HS was not diagnosed in any patient.

In the second validation cohort (IDEAS), all patients had undergone epilepsy surgery, and histopathological confirmation of HS was available in all patients. The control group of this cohort consisted of healthy participants.

### 3.2 Classification of HS in the training (Marburg) cohort

**FIGURE 1:**
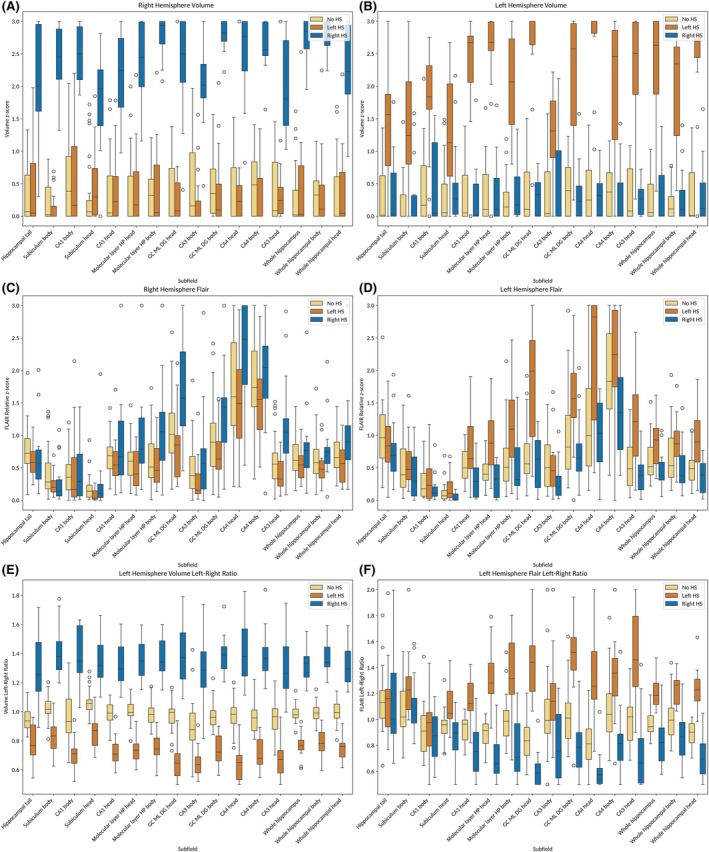
Boxplots of hippocampal subfield features included in the models. (A) Hippocampal subfieldwise inverted volume *z*‐scores as a measure of atrophy on the right and (B) on the left hemisphere. (C) Relative *z*‐scores for fluid‐attenuated inversion recovery (FLAIR) intensity for the right and (D) for the left hippocampus. (E, F) Left–right ratios of volume and FLAIR features. DG, dentate gyrus; GC, granule cell layer; HP, hippocampal; HS, hippocampal sclerosis; ML, molecular layer.

Figure 1 illustrates differences in selected hippocampal subfield features between patients with and without HS (Figure S1 shows all hippocampal subfield features). Feature‐based classifiers demonstrated high classification accuracy across all configurations of image availability in the k‐fold cross‐validation. Highest performance was achieved using MPRAGE + 3D‐FLAIR data (mean accuracy 1.0, CI = .939–1.0); MPRAGE + 2D‐FLAIR (mean accuracy = .950, CI = .772–.999) and MPRAGE alone (mean accuracy = .986, CI = .925–1.0) also showed robust performance (Table 2). Accuracy was chosen as the primary performance measure, as it is possible to compute this parameter for a three‐class classification. Other binary classification metrics (AUC of the ROC, sensitivity, specificity, and F1 score) were calculated for each hemisphere separately. Specificity was high in all configurations, whereas the sensitivity was slightly lower.

**TABLE 2:** Classification results within the Marburg cohort using a 10‐fold cross‐validation procedure (fivefold for 2D FLAIR; see Materials and Methods).

	Measure	Mean	Clopper–Pearson confidence interval
MPRAGE + 3D FLAIR^a^			
All	Accuracy	1.0	.939–1.0
Left	Accuracy	1.0	.921–1.0
	ROC‐AUC	1.0	.992–1.0
	Recall (Sens)	1.0	.782–1.0
	Recall (Spec)	1.0	.884–1.0
	F1 score	1.0	.884–1.0
Right	Accuracy	1.0	.920–1.0
	ROC‐AUC	1.0	.991–1.0
	Recall (Sens)	1.0	.768–1.0
	Recall (Spec)	1.0	.884–1.0
	F1 score	1.0	.877–1.0
MPRAGE + 2D FLAIR^b^			
All	Accuracy	.950	.772–.999
Left	Accuracy	1.0	.794–1.0
	ROC‐AUC	1.0	.940–1.0
	Recall (Sens)	1.0	.541–1.0
	Recall (Spec)	1.0	.692–1.0
	F1 score	1.0	.735–1.0
Right	Accuracy	1.0	.794–1.0
	ROC‐AUC	1.0	.940–1.0
	Recall (Sens)	1.0	.541–1.0
	Recall (Spec)	1.0	.692–1.0
	F1 score	1.0	.735–1.0
MPRAGE only^c^			
All	Accuracy	.986	.925–1.0
Left	Accuracy	.980	.901–1.0
	ROC‐AUC	1.0	.994–1.0
	Recall (Sens)	.90	.727–.999
	Recall (Spec)	1.0	.903–1.0
	F1 score	.90	.851–.999
Right	Accuracy	1.0	.934–1.0
	ROC‐AUC	1.0	.994–1.0
	Recall (Sens)	1.0	.815–1.0
	Recall (Spec)	1.0	.903–1.0
	F1 score	1.0	.903–1.0

*Note*: Accuracy is reported from a three‐class classification procedure (all/left/right vs. no HS), whereas binary classification metrics were evaluated for each hemisphere separately (HS vs. no HS for left and right hemisphere).

Abbreviations: 2D, two‐dimensional; AUC, area under the curve; FLAIR, fluid‐attenuated inversion recovery; MPRAGE, magnetization‐prepared rapid acquisition gradient echo; ROC, receiver operating characteristic; Sens, sensitivity; Spec, specificity.

^a^ Volume (MPRAGE) and 3D FLAIR data used for training.

^b^ Training based on volume (MPRAGE) and 2D FLAIR data.

^c^ Only volume (MPRAGE) data utilized.

### 3.3 External validation

The algorithm was validated in two independent external cohorts, which varied from the training (Marburg) cohort in terms of MRI acquisition protocols. For instance, some FLAIR scans of the first (Berlin) validation cohort had limited brain coverage, covering a smaller field of view that focused on the hippocampi, a condition not included in the classifier's training data. In the first validation cohort, subjects with 2D FLAIR scans were 4.3 times more common than those with 3D FLAIR scans, whereas in the second cohort, 3D FLAIR scans were 3.8 times more common than 2D FLAIR scans.

The validation of model‐based HS detection and lateralization using MPRAGE and FLAIR data demonstrated an accuracy of .889 (CI = .805–.945) in the first (Berlin) validation cohort and of .951 (CI = .902–.980) in the second validation cohort (IDEAS dataset). The accuracies using only MPRAGE data were .811 (CI = .715–.886) in the first and .910 (CI = .851–.951) in the second validation cohort (Table 3). The exact McNemar test was used to compare model performance using MPRARGE and FLAIR scans versus using MPRAGE scans alone. Significantly better predictions were achieved using combined MPRAGE and FLAIR data (Berlin cohort: *p* = .016; IDEAS cohort: *p* = .031).

**TABLE 3:** External validation of the model using either MPRAGE and FLAIR data or MPRAGE data alone of the Berlin and IDEAS cohorts.

Measure	Berlin validation cohort MPRAGE + FLAIR (Clopper–Pearson confidence interval)	Berlin validation cohort MPRAGE (Clopper–Pearson confidence interval)	IDEAS validation cohort MPRAGE + FLAIR (Clopper–Pearson confidence interval)	IDEAS validation cohort MPRAGE (Clopper–Pearson confidence interval)
All				
Accuracy	.889 (.805–.945)	.811 (.715–.886)	.951 (.902–.980)	.910 (.851–.951)
Left				
Accuracy	.920 (.834–.970)	.827 (.722–.904)	.991 (.950–1.0)	.954 (.896–.985)
ROC‐AUC	.905 (.888–.921)	.80 (.777–.821)	.988 (.983–.992)	.958 (.950–.965)
Recall [Sens]	.852 (.663–.958)	.704 (.498–.862)	.976 (.871–.999)	.976 (.871–.999)
Recall [Spec]	.958 (.857–.995)	.896 (.773–.965)	1.0 (.947–1.0)	.941 (.856–.984)
F1 score	.885 (.766–.956)	.745 (.604–.857)	.988 (.933–1.0)	.941 (.868–.981)
Right				
Accuracy	.937 (.845–.982)	.937 (.845–.982)	.942 (.878–.978)	.922 (.853–.966)
ROC‐AUC	.867 (.840–.891)	.867 (.840–.891)	.928 (.917–.938)	.907 (.894–.918)
Recall [Sens]	.733 (.449–.922)	.733 (.449–.922)	.886 (.733–.968)	.857 (.697–.952)
Recall [Spec]	1.0 (.926–1.0)	1.0 (.926–1.0)	.971 (.898–.996)	.956 (.876–.991)
F1 score	.846 (.651–.956)	.846 (.651–.956)	.912 (.818–.967)	.882 (.781–.948)

*Note*: Accuracy is reported from a three‐class classification procedure (all/left/right vs. no HS), and additionally binary classification metrics were evaluated for each hemisphere separately (HS vs. no HS for left and right hemisphere).

Abbreviations: AUC, area under the curve; FLAIR, fluid‐attenuated inversion recovery; MPRAGE, magnetization‐prepared rapid acquisition gradient echo; ROC, receiver operating characteristic; Sens, sensitivity; Spec, specificity.

### 3.4 Comparison to a recently published classifier

To compare our approach to a previous, high‐performing classifier (AID‐HS^35^), a new model was trained on both former validation cohorts (IDEAS and Berlin, *n* = 234). This was necessary because the AID‐HS classifier required knowledge of subject age during evaluation, which was only available for the Marburg cohort (now test cohort).

Cross‐validation of our model using this IDEAS + Berlin (training) data resulted in a mean accuracy of .915 (CI = .871–.947) using MPRAGE and FLAIR data and a mean accuracy of .876 (CI = .827–.915) using only MPRAGE data (Table S5).

Testing of the new model on the unseen Marburg data (*n* = 72) resulted in an accuracy of .972 (CI = .903–.997), and the AID‐HS model achieved an accuracy of .958 (CI = .883–.991) on the same dataset. This slight difference was not significant (McNemar exact test, *p* = 1.0; Table 4).

**TABLE 4:** Testing of the model trained on the Berlin and IDEAS cohorts on the now unseen Marburg cohort ("presented method").

Measure	Presented method (Clopper–Pearson confidence interval)	AID‐HS (Clopper–Pearson confidence interval)
All		
Accuracy	.972 (.903–.997)	.958 (.883–.991)
Left		
Accuracy	.981 (.901–1.0)	.963 (.873–.995)
ROC‐AUC	.986 (.974–.994)	.958 (.940–.972)
Recall [Sens]	1.0 (.815–1.0)	.944 (.727–.999)
Recall [Spec]	.972 (.855–.999)	.972 (.855–.999)
F1 score	.973 (.858–.999)	.944 (.813–.993)
Right		
Accuracy	.981 (.901–1.0)	.981 (.901–1.0)
ROC‐AUC	.972 (.956–.983)	.986 (.974–.994)
Recall [Sens]	.944 (.727–.999)	1.0 (.815–1.0)
Recall [Spec]	1.0 (.903–1.0)	.972 (.855–.999)
F1 score	.971 (.851–.999)	.973 (.858–.999)

*Note*: For comparison, performance metrics of the AID‐HS classifier^35^ on the same cohort are reported. Accuracy is reported from a three‐class classification procedure (all/left/right vs. no HS), and additionally binary classification metrics were evaluated for each hemisphere separately (HS vs. no HS for left and right hemisphere).

Abbreviations: AID‐HS, Automated and Interpretable Detection of Hippocampal Sclerosis; AUC, area under the curve; ROC, receiver operating characteristic; Sens, sensitivity; Spec, specificity.

## 4 DISCUSSION

This study presents a highly accurate and flexible software tool for automated HS detection, using standard MRI sequences commonly available in clinical practice. The algorithm demonstrated robust performance across multiple scenarios of varying field strength and image quality (MPRAGE‐only vs. MPRAGE + 3D or 2D FLAIR). Notably, the software tool maintains highly robust performance even with minimal imaging data.

Our analysis of different imaging protocol combinations revealed already highly accurate predictions using 3D MPRAGE scans alone, with further improvement of performance when additional FLAIR scans were provided.

The robustness of the developed algorithm was validated using two external cohorts that had been scanned independently at different tertiary epilepsy centers using different imaging protocols and scanners. Classification performance remained strong in the validation scenarios, demonstrating the model's generalizability across scanners, protocols, and cohorts. Notably, better results were achieved in the second validation cohort, where more 3D FLAIR scans with whole‐brain coverage were available, supporting the findings of the training scenario.

Comparison of our model with the AID‐HS classifier^35^ revealed no statistically significant difference in performance. For this analysis, a new model was trained on the IDEAS + Berlin data for later testing on the unseen Marburg data (see Materials and Methods). The algorithm showed robust results also in these new validation scenarios.

The exceptional performance on the Marburg dataset, be it during training (Section 3.2) or testing (Section 3.4), may be due to it being a “clean” dataset with radiologically clear cases. Potential implications of this are discussed in the limitations below.

Our results also compare favorably with other existing tools, demonstrating superior sensitivity and specificity in distinguishing HS‐related epilepsy from other forms. Prediction accuracies of our tool were at least comparable to those reported in a study that used additional complex analyses such as cortical folding of the temporal pole.^14^ Although previous research reported promising results in sensitivity and specificity using FLAIR images alone,^10^ our software tool demonstrated slightly better results with the additional benefit of allowing epilepsy with HS to be distinguished from epilepsy without HS, as compared to making the distinction from healthy controls alone. Sensitivity and specificity (compared to healthy controls) reported for commercially available tools were lower than those obtained in the present study (NeuroQuant; sensitivity = 68%, specificity = 90.4%, positive predictive value = 84%, negative predictive value = 79.8%, accuracy = 79.4%^36^).^13^

We developed a clinician‐friendly software tool that performs comprehensive image processing from the raw level onward, requiring only a 3D MPRAGE scan as minimum input, which can be augmented with either one or more 2D FLAIR scans or one 3D FLAIR scan to improve accuracy. For cases of suspected bilateral HS, the tool includes a separate model trained on isolated hemispheres, albeit with slightly lower accuracy (Tables S4 and S6).

The software tool is also available as a dockerized solution and can be run on Linux, MacOS, or Windows operating systems. Its fast runtime and minimal data requirements (no demographic data input, no special input structure like Brain Imaging Data Structure (BIDS) in other tools^35^) make it easy to use.

## 5 LIMITATIONS

A limitation of this study was the relatively small sample size and insufficient histological validation within a relevant fraction of the training (Marburg) cohort. The software tool was, however, validated using two independent validation cohorts with 234 subjects in sum, among whom most of the patients with HS had been confirmed via histopathology.

The high classification performance of both our tool and the AID‐HS classifier on the Marburg dataset indicate that this cohort may constitute a “clean” dataset, consisting of mostly radiologically clear‐cut cases. However, external validation of models trained on these data still showed highly promising performance, indicating model generalizability. We also provide an additional model that had been trained on the larger and less clear‐cut IDEAS + Berlin cohorts, overcoming the limitations of the original training (Marburg) cohort. Prospective, real‐world evaluation of our model is warranted to prove its efficacy under clinical conditions, where the proportion of borderline cases may be higher. In such a setting, the analysis of model performance compared to clinical raters would be required to further clarify the added value of our tool.

## 6 CONCLUSIONS

The developed software tool offers an accurate, automated solution for HS detection using standard clinical MRI scans. Its performance remained robust across different imaging protocols and scanners in two independent, external datasets. Our results, together with previous promising models,^35^ provide converging evidence that automated tools for detection of HS have reached a level where clinical translation is becoming feasible. Future work will focus on model refinement using larger datasets, prospective real‐world model evaluation, and expanding its application to other epilepsy centers.

## AUTHOR CONTRIBUTIONS

**Marcus Belke:** Design of the study; patient acquisition; calculation and statistical evaluation; writing the manuscript. **Felix Zahnert:** Design of the study; patient acquisition; Calculation and statistical evaluation; writing the manuscript. **Mirja Steinbrenner:** External validation; reviewing the manuscript. **Mustafa Halimeh:** External validation; reviewing the manuscript. **Gadi Miron:** External validation; reviewing the manuscript. **Panagiota‐Eleni Tsalouchidou:** Patient acquisition; reviewing the manuscript. **Louise Linka:** Patient acquisition; reviewing the manuscript. **Boris Keil:** Reviewing the manuscript. **Andreas Jansen:** Reference cohort; reviewing the manuscript. **Vincent Möschl:** Histopathological evaluation of training cohort (Marburg); reviewing the manuscript. **André Kemmling:** Neuroradiological evaluation; reviewing the manuscript. **Christopher Nimsky:** Neurosurgery; reviewing the manuscript. **Felix Rosenow:** Acquisition of funding; reviewing the manuscript. **Katja Menzler:** Patient acquisition; reviewing the manuscript. **Susanne Knake:** Design of the study; reviewing the manuscript.

## CONFLICT OF INTEREST STATEMENT

None of the authors has any conflict of interest to disclose. A.K. is a consultant for Phenox, Penumbra, and Stryker. C.N. is a consultant for Brainlab and has received speaker honoraria from Aesculap, BKmedical, and Brainlab. F.R. reports personal fees and nonfinancial support from UCB Pharma; personal fees from Angelini Pharma, Desitin Pharma, Eisai, Jazz Pharma, LMU Munich, Medilearn India, and Roche Pharma; research grants from the German Research Foundation, Bundesministerium für Bildung und Forschung–ERAPerMed Program, European Union (FP7), Hessisches Ministerium für Wissenschaft und Kunst (LOEWE Program), Hessisches Ministerium für Soziales und Integration, Detlev‐Wrobel‐Fonds for Epilepsy Research Frankfurt, Reiss‐Stiftung, Dr. Senckenbergische‐Stiftung, and Ernst Max von Grunelius‐Stiftung; and grants from the Chaja Foundation, Dr. Schär Deutschland, Vitaflo Deutschland, Nutricia Milupa, and Desitin Pharma, Hamburg, outside the submitted work. K.M. has received speaker honoraria/consultancy fees from UCB, Eisai, and Bial. S.K. has received speaker's honoraria from Bial, Eisai, Desitin, Jazz Pharma, Kanso, Merck Serono, UCB, and Zogenix. We confirm that we have read the Journal's position on issues involved in ethical publication and affirm that this report is consistent with those guidelines.

## Supporting information


Data S1.

## Data Availability

Data from the training (Marburg) cohort are available on request from the corresponding author (M.B.). The data are not publicly available due to privacy or ethical restrictions. The data of the first validation cohort are not publicly available. The data of the second validation cohort are freely available and can be downloaded by following a link provided in the original publication.^15^ The code of the software tool as well as the trained models are available on request from the corresponding author (M.B.).
