# Supplementary material for: Automatic detection of hippocampal sclerosis in patients with epilepsy
Source: Epilepsia. 2025 Jun 21;66(10):3852–64. doi: 10.1111/epi.18514 (PMC12605791; doi:10.1111/epi.18514)
Supplement: Supplementary file 1 — Data S1. [file EPI-66-3852-s001.docx]

# Supplementary Material

## 1. MRI Sequences of the training cohort

***Table S1****. Characteristics of 3D MPRAGE Scans (Marburg cohort).*

| **Field Strength (T)** | **Model** | **TE (s)** | **TI (s)** | **TR (s)** | **FA (°)** | **All** | **Control** | **Left HS** | **Right HS** |
| --- | --- | --- | --- | --- | --- | --- | --- | --- | --- |
| 3 | TrioTim | 0.00226 | 0.9 | 1.9 | 9 | 39 | 26 | 5 | 8 |
| 3 | TrioTim | 0.00252 | 0.9 | 1.9 | 9 | 23 | 5 | 9 | 9 |
| 1.5 | Espree | 0.00314 | 1.1 | 2.09 | 15 | 4 | 3 | 1 | 0 |
| 1.5 | Avanto | 0.00308 | 1.1 | 2.09 | 15 | 3 | 1 | 2 | 0 |
| 1.5 | Espree | 0.00314 | 1.1 | 1.97 | 15 | 1 | 1 | 0 | 0 |
| 1.5 | Espree | 0.00314 | 1.1 | 2.17 | 15 | 1 | 0 | 1 | 0 |
| 3 | TrioTim | 0.00215 | 0.8 | 1.44 | 15 | 1 | 0 | 0 | 1 |
|  |  |  |  |  |  | 72 | 36 | 18 | 18 |

***Table S2****. Characteristics of 3D FLAIR Scans (Marburg cohort).*

| **Field Strength (T)** | **Model** | **TE (s)** | **TI (s)** | **TR (s)** | **FA (°)** | **All** | **Control** | **Left HS** | **Right HS** |
| --- | --- | --- | --- | --- | --- | --- | --- | --- | --- |
| 3 | TrioTim | 0.394 | 1.8 | 5 | 120 | 47 | 29 | 9 | 9 |
| 3 | TrioTim | 0.393 | 2.1 | 6 | 120 | 8 | 0 | 4 | 4 |
| 1.5 | Avanto | 0.358 | 2.2 | 6 | 120 | 1 | 0 | 1 | 0 |
| 1.5 | Espree | 0.376 | 2.2 | 6 | 120 | 1 | 1 | 0 | 0 |
| 1.5 | Espree | 0.363 | 1.8 | 5 | 120 | 1 | 0 | 1 | 0 |
| 3 | TrioTim | 0.388 | 1.8 | 5 | 120 | 1 | 0 | 0 | 1 |
|  |  |  |  |  |  | 59 | 30 | 15 | 14 |

***Table S3.*** *Characteristics of coronal 2D FLAIR Scans (Marburg cohort).*

| **Field Strength (T)** | **Model** | **TE (s)** | **TI (s)** | **TR (s)** | **FA (°)** | **All** | **Control** | **Left HS** | **Right HS** |
| --- | --- | --- | --- | --- | --- | --- | --- | --- | --- |
| 3 | TrioTim | 0.093 | 2.5 | 9 | 130 | 13 | 6 | 3 | 4 |
| 1.5 | Espree | 0.087 | 2.5 | 9 | 150 | 4 | 3 | 1 | 0 |
| 1.5 | Avanto | 0.087 | 2.5 | 9 | 150 | 2 | 1 | 1 | 0 |
| 3 | TrioTim | 0.093 | 2.8968 | 14.99 | 130 | 1 | 0 | 0 | 1 |
| 3 | TrioTim | 0.093 | 2.5012 | 9.01 | 130 | 1 | 0 | 1 | 0 |
| 3 | TrioTim | 0.094 | 2.5 | 10 | 120 | 1 | 0 | 0 | 1 |
|  |  |  |  |  |  | 22 | 10 | 6 | 6 |

## 2. Visualization of all hippocampal subfield features

Figure S1 highlights raw features computed for all hippocampal subfields, including those excluded from the training process (hippocampal fissure, fimbria, HATA, presubiculum head/body and parasubiculum).


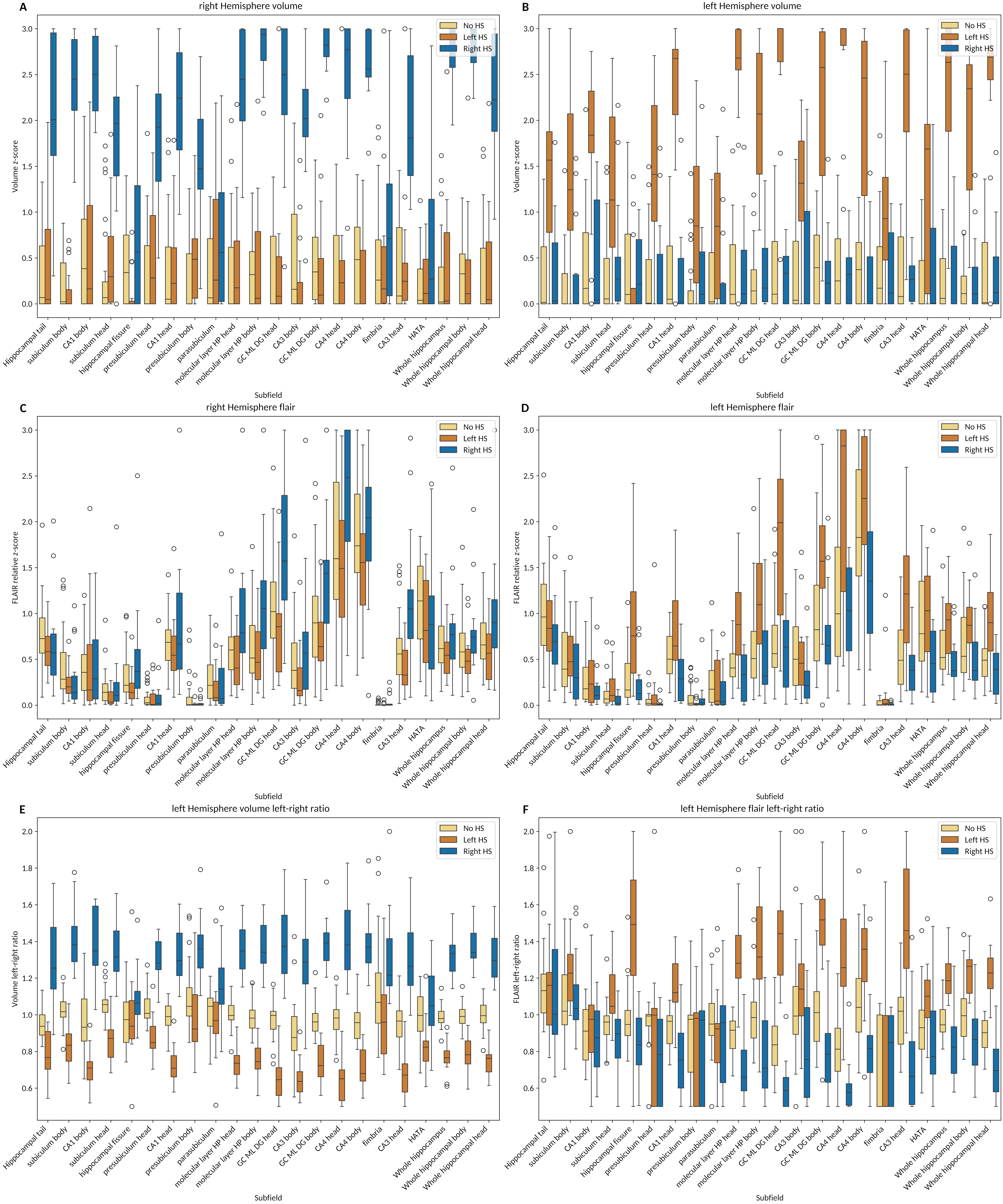
**Figure S1.** Boxplots of all calculated hippocampal subfield features, including those excluded from model training (hippocampal fissure, fimbria, HATA, presubiculum head/body and parasubiculum). A) Hippocampal subfield-wise inverted volume z-scores as a measure of atrophy on the right and B) on the left hemisphere. C) Z-scores for FLAIR intensity for the right and D) for the left hippocampus. E) and F) depict left-right-ratios of volume- and FLAIR features.

## 3. Additional classifier for identification of bilateral hippocampal sclerosis

The classifiers described in the manuscript allow for the detection of unilateral HS. To provide the ability to detect bilateral HS, an additional model was trained. Here, due to the lack of bilateral cases in our cohort, classification was conducted on a within-hemisphere basis, while left-right ratios of hippocampal features were omitted. This approach employed a two-class classification per hemisphere (HS vs. no HS) rather than the three-class classification presented in the main analysis (right HS vs. left HS vs. no HS). The machine-learning procedures remained identical to the main analysis, with binary metrics such as ROC-AUC or Sensitivity reported for each hemisphere. Users can either use this model (with slightly reduced classification accuracy) or the model from the main analysis. Results are summarized in Table S4.

***Table S4****. Classification results on a “per hemisphere”-basis, allowing for theoretical detection of bilateral HS. Depicted are classification results within the Marburg training cohort using a 10-fold (5-fold for 2D FLAIR) cross-validation procedure. A) volume (MPRAGE) and 3D FLAIR data with each hemisphere being evaluated separately. B) Same analysis using volume (MPRAGE) and 2D FLAIR data and C) using volume (MPRAGE) data only.*

|  | **Measure** | **Mean** | **Clopper-Pearson Confidence Interval** |
| --- | --- | --- | --- |
| **A) MPRAGE + 3D FLAIR** |  |  |  |
| **Left** |  |  |  |
|  | Accuracy | 0.930 | 0.817 - 0.986 |
|  | ROC-AUC | 1.0 | 0.992 - 1.0 |
|  | Recall(Sens) | 0.80 | 0.595 - 0.983 |
|  | Recall(Spec) | 0.967 | 0.828 - 0.999 |
|  | F1-Score | 0.780 | 0.726 - 0.978 |
| **Right** |  |  |  |
|  | Accuracy | 1.0 | 0.920 - 1.0 |
|  | ROC-AUC | 1.0 | 0.991 - 1.0 |
|  | Recall(Sens) | 1.0 | 0.768 - 1.0 |
|  | Recall(Spec) | 1.0 | 0.884 - 1.0 |
|  | F1-Score | 1.0 | 0.877 - 1.0 |
| **B) MPRAGE + 2D FLAIR** |  |  |  |
| **Left** |  |  |  |
|  | Accuracy | 1.0 | 0.794 - 1.0 |
|  | ROC-AUC | 1.0 | 0.940 - 1.0 |
|  | Recall(Sens) | 1.0 | 0.541 - 1.0 |
|  | Recall(Spec) | 1.0 | 0.692 - 1.0 |
|  | F1-Score | 1.0 | 0.735 - 1.0 |
| **Right** |  |  |  |
|  | Accuracy | 1.0 | 0.794 - 1.0 |
|  | ROC-AUC | 1.0 | 0.940 - 1.0 |
|  | Recall(Sens) | 1.0 | 0.541 - 1.0 |
|  | Recall(Spec) | 1.0 | 0.692 - 1.0 |
|  | F1-Score | 1.0 | 0.735 - 1.0 |
| **C) MPRAGE only** |  |  |  |
| **Left** |  |  |  |
|  | Accuracy | 0.940 | 0.846 - 0.988 |
|  | ROC-AUC | 1.0 | 0.994 - 1.0 |
|  | Recall(Sens) | 0.850 | 0.653 - 0.986 |
|  | Recall(Spec) | 0.967 | 0.855 - 0.999 |
|  | F1-Score | 0.847 | 0.769 - 0.982 |
| **Right** |  |  |  |
|  | Accuracy | 1.00 | 0.934 - 1.0 |
|  | ROC-AUC | 1.00 | 0.994 - 1.0 |
|  | Recall(Sens) | 1.00 | 0.815 - 1.0 |
|  | Recall(Spec) | 1.00 | 0.903 - 1.0 |
|  | F1-Score | 1.00 | 0.903 - 1.0 |

***Table S5.*** *Classification results within the Berlin and Ideas cohort using a 10-fold cross-validation procedure. A) volume (MPRAGE) and combined 2D and 3D FLAIR data used for training, B) only volume (MPRAGE) data utilized. Accuracies are reported from a three-class classification procedure (“All”, left vs. right vs. no HS), while binary classification metrics were evaluated for each hemisphere separately (“Left” and “Right” for HS vs. no HS, respectively).*

|  | **Measure** | **Mean** | **Clopper-Pearson Confidence Interval** |
| --- | --- | --- | --- |
| **A) MPRAGE + FLAIR** |  |  |  |
| **All** |  |  |  |
|  | Accuracy | 0.915 | 0.871 - 0.947 |
| **Left** |  |  |  |
|  | Accuracy | 0.929 | 0.882 - 0.962 |
|  | ROC-AUC | 0.979 | 0.975 - 0.982 |
|  | Recall(Sens) | 0.90 | 0.799 - 0.958 |
|  | Recall(Spec) | 0.948 | 0.891 - 0.981 |
|  | F1-Score | 0.901 | 0.841 - 0.948 |
| **Right** |  |  |  |
|  | Accuracy | 0.958 | 0.915 - 0.983 |
|  | ROC-AUC | 0.980 | 0.976 - 0.983 |
|  | Recall(Sens) | 0.940 | 0.835 - 0.987 |
|  | Recall(Spec) | 0.965 | 0.914 - 0.991 |
|  | F1-Score | 0.930 | 0.862 - 0.972 |
| **C) MPRAGE only** |  |  |  |
| **All** |  |  |  |
|  | Accuracy | 0.876 | 0.827 - 0.915 |
| **Left** |  |  |  |
|  | Accuracy | 0.908 | 0.856 - 0.945 |
|  | ROC-AUC | 0.926 | 0.920 - 0.931 |
|  | Recall(Sens) | 0.857 | 0.746 - 0.927 |
|  | Recall(Spec) | 0.940 | 0.880 - 0.975 |
|  | F1-Score | 0.869 | 0.803 - 0.924 |
| **Right** |  |  |  |
|  | Accuracy | 0.940 | 0.892 - 0.971 |
|  | ROC-AUC | 0.949 | 0.943 - 0.954 |
|  | Recall(Sens) | 0.840 | 0.709 - 0.928 |
|  | Recall(Spec) | 0.982 | 0.939 - 0.998 |
|  | F1-Score | 0.893 | 0.813 - 0.948 |

***Table S6****. Classification results on a “per hemisphere”-basis, allowing for theoretical detection of bilateral HS. Depicted are classification results within the Ideas and Berlin training cohort using a a 10-fold cross-validation procedure. A) volume (MPRAGE) and pooled 2D and 3D FLAIR data with each hemisphere being evaluated separately. B) using volume (MPRAGE) data only.*

|  | **Measure** | **Mean** | **Clopper-Pearson Confidence Interval** |
| --- | --- | --- | --- |
| **A) MPRAGE + FLAIR** |  |  |  |
| **Left** |  |  |  |
|  | Accuracy | 0.945 | 0.902 - 0.974 |
|  | ROC-AUC | 0.981 | 0.977 - 0.984 |
|  | Recall(Sens) | 0.943 | 0.856 - 0.984] |
|  | Recall(Spec) | 0.947 | 0.891 - 0.981 |
|  | F1-Score | 0.929 | 0.871 - 0.965 |
| **Right** |  |  |  |
|  | Accuracy | 0.939 | 0.892 - 0.971 |
|  | ROC-AUC | 0.995 | 0.992 - 0.996 |
|  | Recall(Sens) | 0.920 | 0.808 - 0.978 |
|  | Recall(Spec) | 0.947 | 0.891 - 0.981 |
|  | F1-Score | 0.903 | 0.827 - 0.952 |
| **B) MPRAGE only** |  |  |  |
| **Left** |  |  |  |
|  | Accuracy | 0.918 | 0.869 - 0.954 |
|  | ROC-AUC | 0.914 | 0.907 - 0.920 |
|  | Recall(Sens) | 0.826 | 0.712 - 0.905 |
|  | Recall(Spec) | 0.974 | 0.926 - 0.995 |
|  | F1-Score | 0.878 | 0.813 - 0.932 |
| **Right** |  |  |  |
|  | Accuracy | 0.939 | 0.892 - 0.971 |
|  | ROC-AUC | 0.945 | 0.939 - 0.951 |
|  | Recall(Sens) | 0.880 | 0.757 - 0.955 |
|  | Recall(Spec) | 0.964 | 0.914 - 0.991 |
|  | F1-Score | 0.896 | 0.820 - 0.950 |

## 4. **Exemplary case**


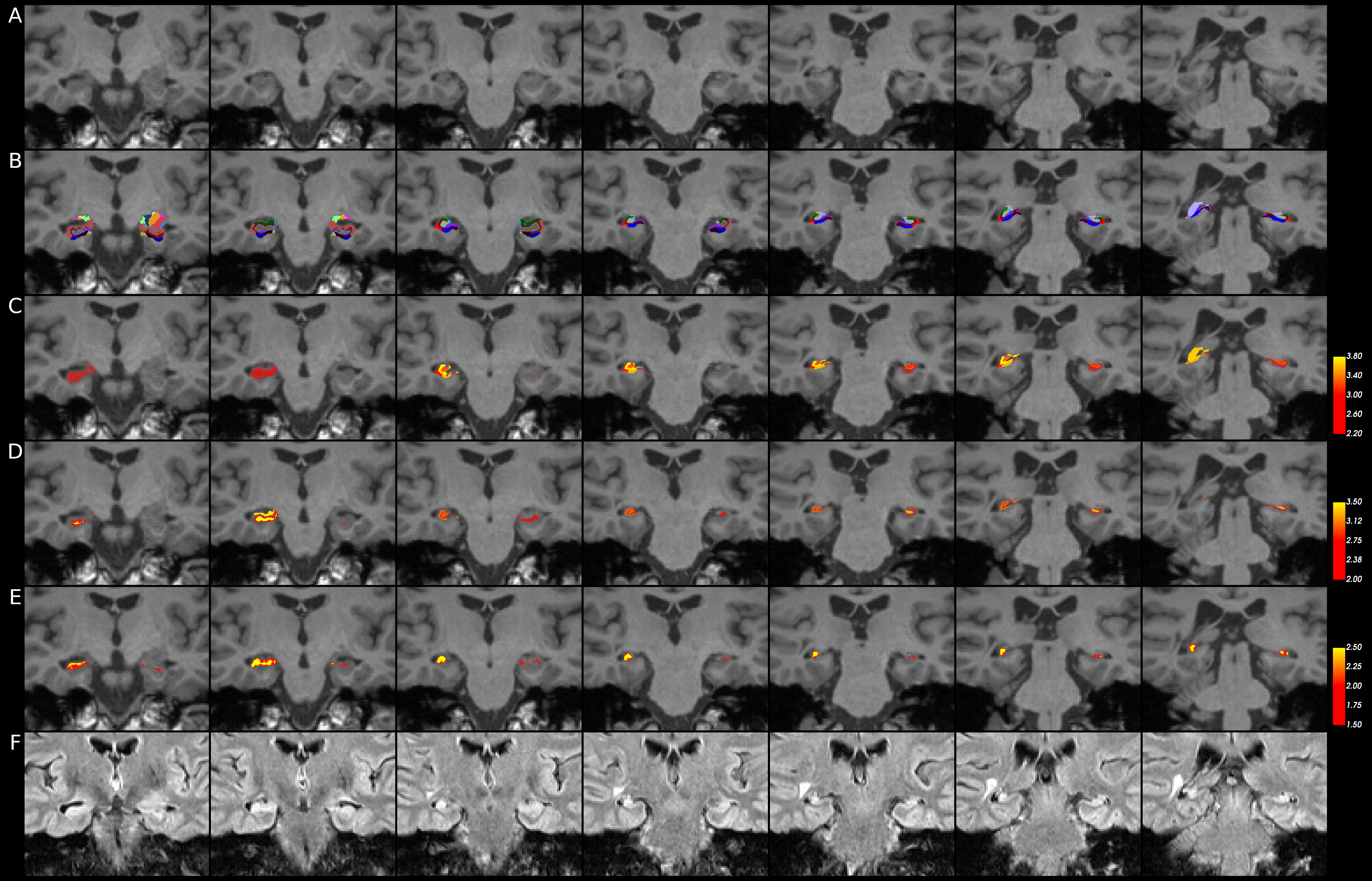
**Figure S2:** MPRAGE and FLAIR slices of a sample subject A) raw MPRAGE image B) hippocampal subfield segmentation C) hippocampal subfield-wise inverted volume z-scores as a measure of atrophy D) hippocampal subfield-wise relative FLAIR z-scores as a measure of hyperintensity. E) z-score of hyperintensity in relation to this subject’s remainder of the cortex for each voxel of the hippocampus. F) raw FLAIR image.
